# Modified multiple marker aneuploidy screening as a primary screening test for preeclampsia

**DOI:** 10.1186/s12884-022-04514-4

**Published:** 2022-03-08

**Authors:** Tianhua Huang, H. Melanie Bedford, Shamim Rashid, Evasha Rasasakaram, Megan Priston, Ellen Mak-Tam, Clare Gibbons, Wendy S. Meschino, Howard Cuckle, Elad Mei-Dan

**Affiliations:** 1 Genetics Program, North York General Hospital, 4001 Leslie Street, Toronto, ON M2K 1E1 Canada; 2 Prenatal Screening Ontario, Better Outcomes Registry & Network (BORN) Ontario, Ottawa, ON Canada; 3 Department of Obstetrics and Gynecology, University of Toronto, Toronto, ON Canada; 4 Department of Paediatrics, University of Toronto, Toronto, ON Canada; 5 Department of Molecular Genetics, University of Toronto, Toronto, ON Canada; 6 Faculty of Medicine, Tel Aviv University, Tel Aviv, Israel; 7 Maternal and Newborn Program, North York General Hospital, Toronto, ON Canada

**Keywords:** Multiple marker screening, Preeclampsia, Gestational hypertension, Preterm birth, Pregnancy-associated plasma protein A, Placental growth factor, Human chorionic gonadotropin, Alpha feto-protein, Unconjugated estriol and Inhibin A

## Abstract

**Background:**

Abnormal levels of maternal biochemical markers used in multiple marker aneuploidy screening have been associated with adverse pregnancy outcomes. This study aims to assess if a combination of maternal characteristics and biochemical markers in the first and second trimesters can be used to screen for preeclampsia (PE). The secondary aim was to assess this combination in identifying pregnancies at risk for gestational hypertension and preterm birth.

**Methods:**

This case-control study used information on maternal characteristics and residual blood samples from pregnant women who have undergone multiple marker aneuploidy screening. The median multiple of the median (MoM) of first and second trimester biochemical markers in cases (women with PE, gestational hypertension and preterm birth) and controls were compared. Biochemical markers included pregnancy-associated plasma protein A (PAPP-A), placental growth factor (PlGF), human chorionic gonadotropin (hCG), alpha feto-protein (AFP), unconjugated estriol (uE3) and Inhibin A. Logistic regression analysis was used to estimate screening performance using different marker combinations. Screening performance was defined as detection rate (DR) and false positive rate (FPR). Preterm and early-onset preeclampsia PE were defined as women with PE who delivered at < 37 and < 34 weeks of gestation, respectively.

**Results:**

There were 147 pregnancies with PE (81 term, 49 preterm and 17 early-onset), 295 with gestational hypertension, and 166 preterm birth. Compared to controls, PE cases had significantly lower median MoM of PAPP-A (0.77 vs 1.10, *p* < 0.0001), PlGF (0.76 vs 1.01, *p* < 0.0001) and free-β hCG (0.81 vs. 0.98, *p* < 0.001) in the first trimester along with PAPP-A (0.82 vs 0.99, *p* < 0.01) and PlGF (0.75 vs 1.02, *p* < 0.0001) in the second trimester. The lowest first trimester PAPP-A, PlGF and free β-hCG were seen in those with preterm and early-onset PE. At a 20% FPR, 67% of preterm and 76% of early-onset PE cases can be predicted using a combination of maternal characteristics with PAPP-A and PlGF in the first trimester. The corresponding DR was 58% for gestational hypertension and 36% for preterm birth cases.

**Conclusions:**

Maternal characteristics with first trimester PAPP-A and PlGF measured for aneuploidy screening provided reasonable accuracy in identifying women at risk of developing early onset PE, allowing triage of high-risk women for further investigation and risk-reducing therapy. This combination was less accurate in predicting women who have gestational hypertension or preterm birth.

## Background

Maternal multiple marker screening for fetal aneuploidy is part of routine prenatal care. Over the past decades, multiple marker screening tests, comprising of biochemical markers and the ultrasound marker nuchal translucency (NT), have evolved from second to first trimester screening. In recent years, there has been an increasing uptake of cell free fetal DNA (cffDNA) in aneuploidy screening. For screening programs that use a contingent cffDNA approach, multiple marker screening has been used as a first-tier screen to identify pregnancies at increased risk of fetal aneuploidy followed by cffDNA or diagnostic testing [1, 2]. Maternal biochemical markers used in multiple marker screening have long been associated with adverse pregnancy outcomes such as preeclampsia (PE), preterm birth and intrauterine growth restriction (IUGR) [3, 4]. Results from recent studies have shown that about 65% of early-onset PE can be predicted using first trimester maternal serum placental growth factor (PlGF), pregnancy-associated plasma protein-A (PAPP-A) and maternal demographics and history at a false positive rate (FPR) of 5% [5]. The accuracy increased to 90% for early-onset PE and 80% for preterm PE with the addition of mean arterial blood pressure (MAP) and uterine artery pulsatility index (UTPI) at a 5% FPR [5]. Moreover, studies have shown that the administration of nightly low-dose aspirin before 16 weeks of gestation, to women who are identified as high-risk for PE, can prevent about 90% of early-onset PE cases [6]. Second trimester biochemical markers were also found to be helpful in identifying women at risk of developing PE, allowing for close surveillance and a timely intervention [7–9].

The ideal screening test for PE should include maternal characteristics, medical and family history, and biochemical and biophysical markers [10]. In reality, biophysical markers such as MAP and especially UTPI might not be readily accessible, especially to women in remote areas. Since PlGF has recently been added to some screening programs to improve the accuracy of first trimester aneuploidy screening, maternal characteristics (questionnaire) combined with biochemical markers (blood test) could be used as a first-tier test to identify women for secondary MAP (physical test) and UTPI (ultrasound scan), allowing for the expansion of current aneuploidy screening programs to include PE screening. A contingent PE screening approach would not only be cost effective but also easy to implement, as it requires minimal change to current aneuploidy screening. Women who are identified as high-risk in the first trimester can be triaged for MAP and UTPI or be considered for prophylactic therapy (e.g. Aspirin) initiated before 16 weeks of gestation to reduce the risk for developing PE [6, 10]. The results from the second trimester screening might be useful for modifying the first trimester risk, assessing the effectiveness of preventive prophylactic treatment and optimizing the management of at-risk pregnancies. Our study assessed the first stage of this contingent approach by using maternal characteristics with first and/or second trimester biochemical markers to predict PE, in addition to other adverse pregnancy outcomes including gestational hypertension and preterm birth.

## Methods

### Study population

A retrospective case-control study was carried out using frozen residual serum samples from women who had undergone multiple marker screening and delivered at a tertiary center in Toronto, Ontario, Canada between January 1, 2014 and October 31, 2017.

A combined dataset was created by linking multiple marker screening records with maternal and newborn records using patient identifiers and expected date of delivery (EDD). Women who had a first and/or second trimester serum sample available were included in this study. The exclusion criteria included multiple pregnancies, pregnancies with a fetal anomaly, and those with unavailable or unmeasurable residual samples. Cases were identified from maternal records of women who had PE, gestational hypertension, or preterm birth. PE cases were further classified as preterm (women who developed PE and gave birth at < 37 weeks of gestation), early-onset (women who developed PE and gave birth at < 34 weeks of gestation) or all PE. Women with early-onset PE were included in the subgroup of preterm PE cases. Early-onset, preterm and term PE cases were included in the group of all PE cases. Each affected pregnancy was matched with three controls by date of blood sample draw (within 30 days), gestational age at first blood sample draw (within 7 days), maternal age (within 5 years), maternal ethnicity and amount of residual sample. Controls were selected from singleton pregnancies that had a live full term birth and were not complicated by fetal anomalies, PE, pregnancy hypertension, preterm birth, preterm premature rupture of the membranes and fetal growth restriction.

During the study period, most of the women were screened using integrated prenatal screening. Others were screened using first trimester screening, enhanced first trimester screening or second trimester serum screening (QUAD). Integrated prenatal screening consisted of NT and PAPP-A measured between 11 + 0 and 13 + 6 weeks, and alpha fetoprotein (AFP), unconjugated estriol (uE3) and intact human chorionic gonadotropin (hCG) measured between 15 + 0 and 18 + 6 weeks. Integrated prenatal screening results were reported after the second blood test was completed. First trimester screening consisted of NT, PAPP-A and free β-hCG measured between 11 + 0 and 13 + 6 weeks. Enhanced first trimester screening was introduced in April 2016 which includes all markers used for first trimester screening and at the same gestational window with the addition of PlGF and AFP. Second trimester QUAD consisted of AFP, uE3, total hCG and inhibin A measured between 15 + 0 to 20 + 6 weeks. As all first trimester samples had PAPP-A measured and all second trimester samples had AFP, uE3 and total hCG measured, the concentrations of these markers from routine aneuploidy screening records were used in this study. Other markers were measured using frozen residual samples. In addition, first trimester inhibin A and second trimester PAPP-A and PlGF that have not been used for aneuploidy screening were also measured using frozen residual samples for this study.

### Biochemical assays

All samples were processed and assayed in a local genetic laboratory. Frozen serum samples had been stored at -20 °C immediately after an initial assay for routine screening and at -70 °C four weeks later for long term storage. All except 33 samples had one freeze-thaw cycle, and none had been used in other studies. Samples having two freeze-thaw cycles were from women who had originally enrolled for integrated prenatal screening but did not provide a second sample in time to complete the test. Therefore, the first sample was converted to first trimester screening and was measured for free β-hCG. All assays were performed on the AutoDELFIA analyzer (PerkinElmer). The AutoDELFIA routine dictates a solid phase, two-site fluorometric assay using two monoclonal antibodies directed against two separate antigenic sites on the molecule. One antibody is labelled with a fluorescent marker, and after incubation, the europium or samarium forms fluorescent chelates. The fluorescence is proportional to the concentration of analyte in the sample.

### Statistical analysis

The differences in the median of continuous variables including maternal age at EDD, maternal weight and body mass index (BMI), gestational age at birth, gestational age at first and second sample among cases and controls were tested using Mann-Whitney U-tests. Categorical variables such as ethnicity, pregnancy outcome, parity, gravidity, chronic hypertension, Type 1 diabetes, Type 2 diabetes, smoking and in vitro fertilization (IVF) were compared using chi-square or Fisher’s exact tests.

The concentrations of biochemical markers in this study were expressed as Multiple of the Median (MoM) calculated by dividing marker concentrations by the expected median values at a particular gestational age. The expected median was generated by weighted regression analysis on marker concentrations of all the controls. The MoM values were then adjusted for maternal weight and were further corrected for ethnicity, parity and smoking status where applicable. The adjustment factors for ethnicity, parity and smoking status were generated based on MoM values calculated from the current study. As routinely performed in our multiple marker aneuploidy screening laboratory, for pregnancies with missing maternal weight, a weight was imputed based on ethnic-specific median weight calculated from the study population. No adjustment was made for pregnancies with missing ethnicity, parity, smoking, insulin dependent diabetes mellitus (IDDM) or IVF status. For example, a pregnancy with missing ethnicity and smoking status was treated as a Caucasian non-smoker. Multiple of the median values of markers that were taken from the routine screening records were already adjusted for ethnicity, parity and smoking status using factors that were implemented in the screening software Alpha 8.0 (Logical Medical Systems Ltd, London). All analyses going forward were based on adjusted MoM values, unless otherwise specified. To assess if there were significant variations in biochemical markers in pregnancies with adverse outcomes, median MoM values of biochemical markers from cases and controls were compared using Mann-Whitney U-tests.

Of women who had both the first and second trimester serum samples available, a subsequent data set was created to assess the change in biochemical marker concentrations and MoM values between the second and first trimester samples in the case and control groups. The pair-wise changes were calculated by subtracting the value of the first trimester sample from the second trimester sample. The median changes in cases and controls were compared using Mann-Whitney U-tests.

Logistic regression was used to assess if the risk of developing PE, gestational hypertension, and preterm birth can be predicted using a combination of maternal biochemical markers and maternal characteristics. Logistic regression analysis with backward selection was run with all first trimester biochemical markers to determine the best predictive models for each case group using only first trimester markers. This was repeated using second trimester biochemical markers. For models with maternal characteristics, an initial logistic regression with backward selection was run to identify significant maternal characteristics for each case group. Varying biochemical marker combinations were then added to significant maternal characteristics to develop predictive models with a combination of maternal characteristics and biochemical markers in each case group. Receiver operating characteristic (ROC) curves were created to determine detection rates (DR) at a 5%, 10%, and 20% FPR, respectively. The positive and negative likelihood ratios (LR) at each FPR were also estimated. All statistical analysis was carried out using SAS 9.4.

The study was approved by the Research Ethics Board of North York General Hospital on May 17, 2017. All methods were performed in accordance with the relevant guidelines and regulations (Declaration of Helsinki).

## Results

In total, 608 cases and 1,815 controls were identified from 15,640 singleton pregnancies. The case group included 147 pregnancies with PE (81 term, 49 preterm and 17 early-onset), 295 with gestational hypertension, and 166 preterm birth. All but eight cases were matched to three controls. Eight cases had 1-2 controls because the control samples were either unusable or unavailable. Samples were available from both the first and second trimesters for 459 cases and 1358 controls.

Table 1 compares the maternal characteristics, medical history, and pregnancy outcomes among case and control groups. Maternal age and ethnicity were similar among case and control groups. Women with PE delivered two weeks earlier, and those with gestational hypertension delivered one week earlier than the control group. The PE group included a greater proportion of women with a history of chronic hypertension, higher BMI and of nulliparous women compared to controls*.* Live birth pregnancies were lowest among preterm birth cases (72%), followed by early-onset PE (94%), preterm PE (98%), and all PE cases (99%). Median gestational age was 88 days for first trimester samples and 114 days for second trimester samples in both case and control groups. There were more nulliparous women in the case groups. The smoking status was similar in cases and controls. A greater proportion of IVF pregnancies was seen in the case group with gestational hypertension.

**Table 1:** Maternal characteristics, medical history, and pregnancy outcomes among case and control groups

Maternal Characteristics	Preeclampsia						Gestational hypertension (*n* = 295)		Preterm birth (*n* = 166)		Controls (*n* = 1815)	
	**All (*n* = 147)**		**Preterm (*n* = 49)**		**Early-onset (*n* = 17)**							
	***n***	**Median** ^**(5th,95th percentile)**^ **/Proportion(%)**	***n***	**Median** ^**(5th,95th percentile)**^ **/ Proportion (%)**	***n***	**Median** ^**(5th,95th percentile)**^ **/ Proportion(%)**	***n***	**Median** ^**(5th,95th percentile)**^ **/ Proportion(%)**	**n**	**Median** ^**(5th,95th percentile)**^ **/ Proportion(%)**	**n**	**Median** ^**(5th,95th percentile)**^ **/ Proportion(%)**
**Maternal age, years**	147	34.1^NS^ (26.5-40.9)	49	34.9^NS^ (26.0-42.8)	17	34.4^NS^ (28.1-41.6)	295	33.5^NS^ (26.4-41.4)	166	33.9^NS^ (24.9-41.7)	1815	33.5 (26.3-39.9)
**Maternal weight, kg**	146	66.0^*^ (48.6-113.2)	49	65.4^%^ (47.7-98.6)	17	65.5^NS^ (42.3-97.7)	282	71.5^*^ (50.8-110.0)	161	60.5^NS^ (45.5-92.7)	1772	60.5 (46.8-88.5)
**Maternal BMI, kg/M2**	124	25.7^*^ (18.7-43.1)	37	25.2^%^ (18.6-36.0)	13	24.8^NS^ (17.9-32.4)	264	26.3^*^ (19.5-38.5)	134	23.3^NS^ (17.6-35.3)	1560	22.4 (17.9-32.9)
**Gestational age at birth, weeks**	147	37.0^*^ (32.0-40.0)	49	35.0^*^ (28.0-36.0)	17	33.0^*^ (23.0-33.0)	295	38.0^*^ (36.0-40.0)	166	31.0^*^ (20.0-33.0)	1815	39.0 (37.0-41.0)
**Gestational age 1**^**st**^** sample scan, days**	143	88.0^NS^ (81.0-93.0)	48	87.0^NS^ (81.0-93.0)	17	87.0^NS^ (81.0-93.0)	286	88.0^NS^ (82.0-92.0)	158	87.0^NS^ (81.0-93.0)	1769	88.0 (82.0-93.0)
**Gestational age 2**^**nd**^** sample scan, days**	125	114.0^NS^ (107.0-128.0)	43	114.0^NS^ (107.0-131.0)	15	117.0^NS^ (107.0-134.0)	223	114.0^NS^ (107.0-130.0)	128	115.0^NS^ (106.0-129.0)	1404	114.0 (107.0-128.0)
**Ethnicity**												
**Caucasian**	36	24.5^NS^	8	16.3^NS^	< 6	-^NS^	111	37.6^NS^	50	30.1^NS^	589	32.5
**Asian**	85	57.8	35	71.4	12	70.6	154	52.2	90	54.2	983	54.2
**Black**	16	10.9	< 6	-	0	0	19	6.4	11	6.6	137	7.5
**Other/** **Unknown**	10	6.8	< 6	-	< 6	-	11	3.7	15	9.0	106	5.8
**Medical History**												
**Chronic hypertension**	40	27.2^*^	11	22.4^*^	< 6	-^*^	20	6.8^*^	0	0	< 6	-
**Diabetes Type 1**	< 6	^NS^	0	0	0	0	0	0	< 6	-^NS^	< 6	-
**Diabetes** **Type 2**	< 6	-^%^	0	0	0	0	< 6	-^NS^	0	0	6	0.33
**APS/SLE**	0	0	0	0	0	0	< 6	-^%^	< 6	-^NS^	0	0
**Smoking**	< 6	-^NS^	0	0	0	0	10	3.4^NS^	< 6	-^NS^	31	1.7
**Gravidity**^**a,b**^												
**1**	65	45.1^^^	22	46.8^%^	8	47.1^NS^	116	40^^^	51	32.9^NS^	534	30.0
**Parity**^**b, c**^												
**Nulliparous**	90	62.5^*^	27	57.4^%^	9	52.9^NS^	164	56.6^*^	80	51.6 ^¶^	729	41.0
**IVF**	< 6	-^NS^	< 6	-^NS^	0	0	11	3.7 ^¶^	< 6	-^NS^	25	1.4
**Pregnancy Outcome **^**d**^												
**Live Birth**	146	99.3^NS^	48	98.0^%^	16	94.1 ^¶^	295	100	120	72.3^*^	1815	100

^*^p < 0.0001; ^ *p* < 0.001; ¶ *p* < 0.01; % *p* < 0.05; NS Not significant. The significance levels of the differences are reported in respect to the control group

Comparison between case and control groups: Chi-Square or Fisher’s exact test for categorical variables; Mann-Whitney U test for continuous variables

BMI, Body Mass Index

^a^Gravidity is a binary variable categorized as 1 or > 1

^b^Parity and Gravidity were missing among cases and controls: 3 missing among all preeclampsia cases(*n* = 144), 2 missing among preterm preeclampsia cases (*n* = 47), 5 missing among gestational hypertension cases(*n* = 290), 11 missing among preterm birth cases(*n* = 155), and 35 missing among controls(*n* = 1780)

^c^Parity is a binary variable categorized as nulliparous or parous

^d^ Pregnancy Outcome is a binary variable categorized as live birth or stillbirth/pregnancy loss

Table 2 gives the median and interquartile range of MoM of first trimester and second trimester biochemical markers seen in case and control groups. The median MoM of first trimester PAPP-A, PlGF, and free-β hCG were significantly reduced among cases compared to controls. Of women who developed PE, the lowest median MoM of PAPP-A and PlGF were seen in those with preterm PE (0.63 and 0.73), and early-onset PE (0.54 and 0.72), respectively. First trimester median MoM values of AFP increased in the all PE, gestational hypertension and preterm birth groups. Free-β hCG decreased in the PE, gestational hypertension and preterm birth groups. No change in the first trimester inhibin A median MoM was observed in case groups.

**Table 2:** Median multiple of the medians (MoMs) of biochemical markers among case and control groups

Marker	Tested	Preeclampsia						Gestational hypertension		Preterm birth		Controls	
		**All**		**Preterm**		**Early-onset**							
		***n***	**Median MoM (IQR)**	***n***	**Median MoM (IQR)**	***n***	**Median MoM (IQR)**	***n***	**Median MoM (IQR)**	***n***	**Median MoM (IQR)**	***n***	**Median MoM (IQR)**
***First trimester (T1)***													
PAPP-A	Routine^a^	141	0.77 ^*^ (0.54,1.17)	48	0.63 ^*^ (0.49,0.96)	17	0.54 ^¶^ (0.44,0.85)	283	0.93^*^ (0.61,1.30)	157	0.86 ^^^ (0.59,1.42)	1746	1.10 (0.74,1.59)
free-β hCG	Study^b^	141	0.81 ^^^ (0.53,1.13)	48	0.82^%^ (0.52,1.12)	17	0.81 ^NS^ (0.52,1.12)	284	0.88^%^ (0.63,1.30)	157	0.88 ^NS^ (0.60,1.31)	1751	0.98 (0.66,1.51)
PlGF	Study	141	0.76 ^*^ (0.62,1.09)	48	0.73 ^*^ (0.57,0.97)	17	0.72 ^^^ (0.51,0.79)	284	0.92 ^*^ (0.69,1.18)	157	0.91 ^^^ (0.70,1.15)	1751	1.01 (0.80,1.31)
AFP	Study	141	1.16 ^¶^ (0.86,1.53)	48	1.17 ^NS^ (0.87,1.54)	17	0.92 ^NS^ (0.86,1.49)	284	1.11 ^^^ (0.87,1.46)	157	1.27 ^*^ (0.90,1.64)	1751	1.00 (0.78,1.33)
Inhibin A	Study	141	1.03 ^NS^ (0.79,1.41)	48	1.07 ^NS^ (0.83,1.53)	17	0.98 ^NS^ (0.78,1.50)	284	1.08 ^NS^ (0.79,1.46)	157	0.95 ^NS^ (0.72,1.36)	1751	1.00 (0.77,1.35)
***Second trimester (T2)***													
AFP	Routine	126	1.03 ^NS^ (0.83,1.28)	43	1.01 ^NS^ (0.83,1.25)	15	1.01 ^NS^ (0.83,1.28)	223	1.04 ^NS^ (0.83,1.28)	130	1.18 ^*^ (0.94,1.47)	1402	1.01 (0.83,1.22)
uE_3_	Routine	91	1.00 ^NS^ (0.80,1.15)	31	0.92^%^ (0.74,1.08)	10	0.74 ^NS^ (0.67,1.06)	163	1.01 ^NS^ (0.89,1.16)	101	0.98 ^NS^ (0.83,1.15)	1056	1.01 (0.86,1.18)
hCG	Routine	91	1.17^%^ (0.89,1.79)	31	1.30^%^ (1.01,1.97)	10	1.62 ^¶^ (1.27,1.97)	163	1.14 ^NS^ (0.85,1.63)	101	1.09 ^NS^ (0.81,1.67)	1056	1.06 (0.75,1.48)
PAPP-A	Study	110	0.82 ^¶^ (0.57,1.26)	36	0.80^%^ (0.53,1.09)	13	0.87 ^NS^ (0.55,1.26)	189	0.90 ^NS^ (0.60,1.37)	97	0.98 ^NS^ (0.60,1.35)	1140	0.99 (0.68,1.41)
PlGF	Study	113	0.75 ^*^ (0.53,1.08)	37	0.74 ^*^ (0.42,0.88)	13	0.42^*^ (0.28,0.74)	197	0.89 ^¶^ (0.66,1.23)	103	0.79 ^^^ (0.61,1.21)	1187	1.02 (0.76,1.37)
Inhibin A^c^	Routine	6	1.45^%^ (1.19,1.74)	< 6	1.51 ^NS^ (-)	0	-	12	0.95 ^NS^ (0.76,1.21)	9	1.36 ^NS^ (0.98,2.12)	75	0.98 (0.73,1.40)

^*^p < 0.0001; ^^^ *p* < 0.001; ^¶^ *p* < 0.01; ^%^ *p* < 0.05; ^NS^ Not significant. The significance levels of the differences are reported in respect to the control group

MoM: Multiple of the median; IQR: Interquartile range

*PAPP-A* pregnancy-associated plasma protein A, *hCG* human chorionic gonadotropin, *PlGF* placental growth factor, *AFP* alpha feto-protein, *uE3* unconjugated Estriol

^a^Routine- marker measurement from routine screening tests

^b^Study- marker (re)/measured specifically for this study

^c^The IQR of inhibin A for preterm PE and early-onset PE cannot be computed due to small sample size

In the second trimester, median MoM values of PlGF were lower in all PE, gestational hypertension and preterm birth groups compared to controls. MoM values of PAPP-A were lower in the PE group but not in the gestational hypertension and preterm birth groups. Median MoM of hCG was higher in PE groups. The lowest median MoM of PlGF (0.42) and PAPP-A (0.80) was seen in women with early-onset PE and preterm PE, respectively. Median MoM of PlGF was lower and AFP was higher in women with preterm birth, compared to controls.

**Fig. 1:**
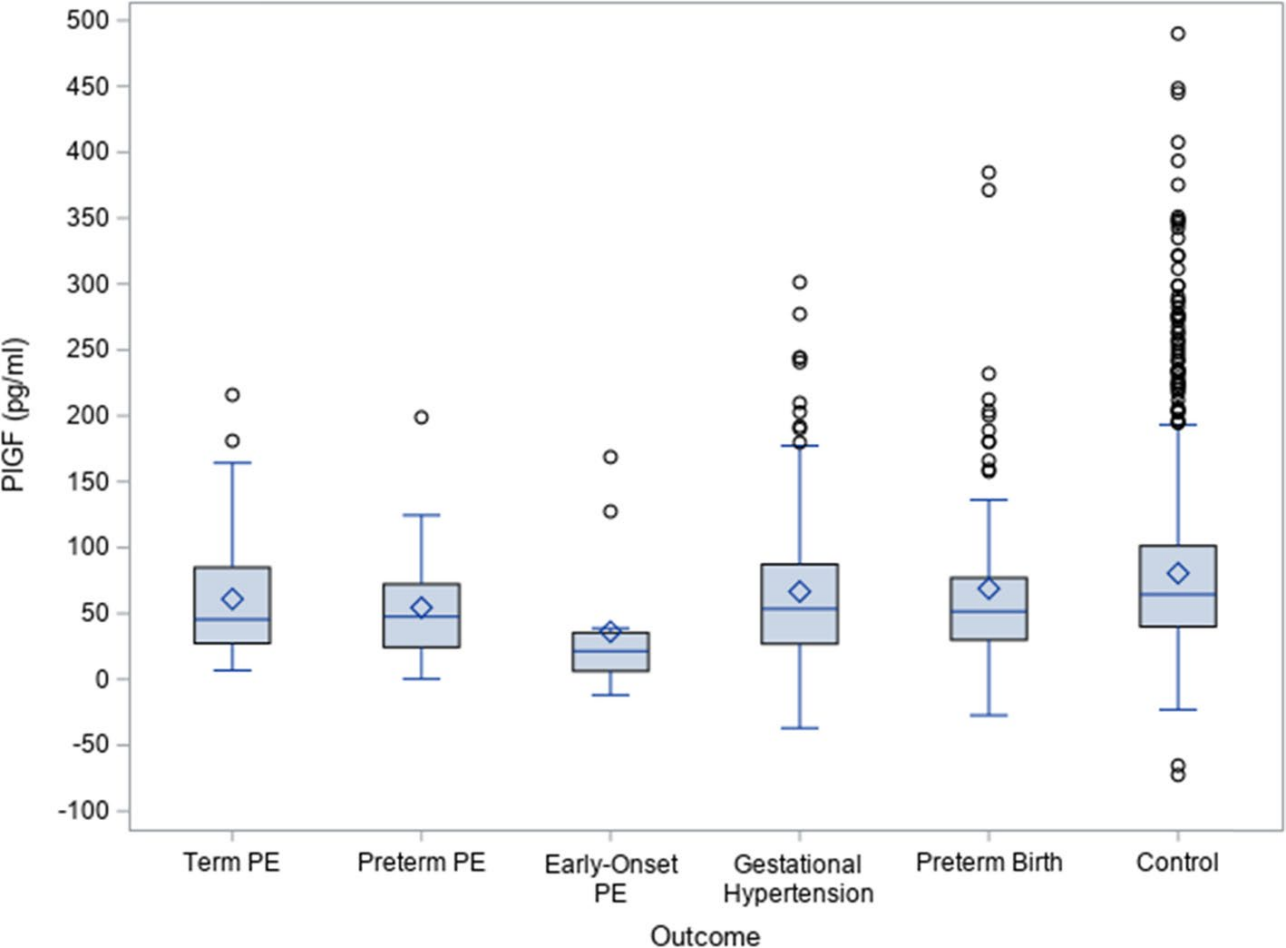
Box and whisker plots for changes in PlGF concentration between the first and second trimester samples among preeclampsia (PE), gestational hypertension, preterm birth cases and controls. Notes: The ends of the box are the upper and lower quartiles of the change. The horizontal line inside the box marks the median change. The two vertical lines outside the box are the whiskers extending to the greatest and smallest changes. The white circles indicate the outliners and the diamond represents the mean change

Figure 1 illustrates the increase in PlGF concentrations between first and second trimester samples; revealing smaller increases among all PE (43.70 pg/ml), preterm PE (32.85 pg/ml), early-onset PE (21.40 pg/ml), and preterm birth cases (51.50 pg/ml) compared to controls (64.40 pg/ml, *p* < 0.01) (Fig. 1). Trends among other biomarkers were not statistically or clinically significant.

Table 3 gives the coefficients of logistic regressions which indicate the degree of the associations between biochemical markers, maternal characteristics and the risk of developing PE, gestational hypertension, and preterm birth. When maternal characteristics were not included in the model, first and second trimester PlGF were associated with all PE, preterm PE and early-onset PE. First trimester PAPP-A, free-β hCG and inhibin A were associated with all PE and preterm PE. When maternal characteristics were included in the model, the associations between PlGF and all PE, preterm PE and early-onset PE remained statistically significant.

**Table 3:** Logistic regression coefficient and standard error for the prediction of adverse pregnancy outcomes using maternal characteristics and biochemical markers

Marker	Preeclampsia						Gestational hypertension		Preterm birth	
	**All**		**Preterm**		**Early-onset**					
	**Coefficient**	**SE**	**Coefficient**	**SE**	**Coefficient**	**SE**	**Coefficient**	**SE**	**Coefficient**	**SE**
***T1 Biochemical Markers***^***a***^										
Intercept	-1.1692^¶^	0.3565	-1.1523NS	0.5985	-1.6729%	0.7381	-1.1012*	0.2421	-1.9447*	0.3125
PAPP-A	-0.7193^^^	0.1853	-1.5537*	0.3874	-	-	-0.4694*	0.1200	-0.3024%	0.1464
free-β hCG	-0.4488^¶^	0.1551	-0.6470%	0.2718	-	-	-0.2657¶	0.1020	-	-
PlGF	-0.9420^^^	0.2780	-1.3867¶	0.5224	-3.3491^	0.9404	-0.3503%	0.1767	-0.5498%	0.2334
AFP	0.1868^%^	0.0898	-	-	-	-	-	-	0.3657^	0.1094
Inhibin A	0.5241^^^	0.1566	0.8701^	0.2274	-	-	0.4195¶	0.1290	-	
***T2 Biochemical Markers***^***b***^										
Intercept	-1.5231^*^	0.3796	-2.5203^*^	0.6174	-2.8837^¶^	1.0162	-2.2967*	0.2990	-3.9344^*^	0.3311
AFP	-	^−^	-	-	-	-	0.7503^^^	0.2113	1.2020^*^	0.2441
uE_3_	-	^−^	-	-	-	-	-	-	-	^−^
hCG	0.5895^*^	0.1483	0.5332^¶^	0.1899	0.6212^%^	0.2678	-	-	-	^−^
PAPP-A	-0.6405^%^	0.2501	-	-	-	-	-0.3987^%^	0.1841	-	^−^
PlGF	-1.0464^^^	0.3104	-1.9338^¶^	0.6259	-3.1643^%^	1.2555	-	-	-	^−^
***Maternal Characteristics***^***c***^** + *T1 PlGF* + *T1 PAPP-A***										
Intercept	-6.9419^*^	1.0096	-5.2600^¶^	1.6327	-5.7611^%^	2.4117	-6.2080^*^	0.7445	-1.7145^*^	0.2782
PlGF	-0.9925^^^	0.2829	-1.7875^¶^	0.5428	-2.8433^¶^	0.9518	-0.3273^NS^	0.1913	-0.4626^NS^	0.2366
PAPP-A	-0.9265^*^	0.1927	-1.5843^*^	0.4034	-1.1291^NS^	0.6044	-0.6066^*^	0.1303	-0.4302^¶^	0.1575
***Maternal Characteristics***^***c***^** + *All T1 Biochemical Markers ***^***a***^										
Intercept	-7.4496^*^	1.0422	-5.9671^^^	1.7274	-6.2326^%^	2.5740	-6.5085^*^	0.7625	-2.0900^*^	0.3673
PAPP-A	-0.9335^*^	0.1960	-1.6698^*^	0.4025	-1.1970^%^	0.5981	-0.6177^*^	0.1337	-0.3836^%^	0.1607
free-β hCG	-0.3955^%^	0.1607	-0.5637^%^	0.2787	-0.2477^NS^	0.3872	-0.1426^NS^	0.1038	-0.0709^NS^	0.1302
PlGF	-0.7713^¶^	0.2817	-1.2278^%^	0.5345	-2.4894^%^	0.9844	-0.2676^NS^	0.1912	-0.4342^NS^	0.2397
AFP	0.2180^%^	0.1004	0.1505^NS^	0.2163	-0.0942^NS^	0.4392	0.1697^%^	0.0863	0.3204^¶^	0.1102
Inhibin A	0.4649^¶^	0.1652	0.8114^^^	0.2419	0.5278^NS^	0.3822	0.2345^NS^	0.1443	0.0016^NS^	0.1835
***Maternal Characteristics***^***c***^** + *All T2 Biochemical Markers ***^***b***^										
Intercept	-5.5285^*^	1.3954	-1.1855^NS^	2.3472	-3.1794^NS^	3.9327	-7.2465^*^	1.1403	-2.9484^*^	0.7141
AFP	-0.4117^NS^	0.3438	-0.7740^NS^	0.5826	-1.1304^NS^	1.1195	0.4014^%^	0.2567	0.9651^¶^	0.3051
uE_3_	-0.4744^NS^	0.5340	-0.9717^NS^	0.9421	-1.9243^NS^	1.6075	-0.0260^NS^	0.4512	-0.5001^NS^	0.5564
hCG	0.6030^^^	0.1700	0.9158^^^	0.2644	0.7432^NS^	0.4389	0.2084^NS^	0.1527	0.1450^NS^	0.2074
PAPP-A	-0.8083^¶^	0.2598	-0.8946^%^	0.4279	0.3250^NS^	0.5517	-0.6117^¶^	0.2133	-0.5368 ^NS^	0.3105
PlGF	-1.0086^¶^	0.3186	-1.4560^%^	0.5983	-3.0874^%^	1.2967	-0.3119^NS^	0.2161	-0.3736^NS^	0.2901

^*^p < 0.0001; ^^^ *p* < 0.001; ^¶^ *p* < 0.01; ^%^ *p* < 0.05; ^NS^ Not significant, The significance levels of the differences are reported in respect to the control group

*SE* Standard error

*PAPP-A* pregnancy-associated plasma protein A, *hCG* human chorionic gonadotropin, *PlGF* placental growth factor, *AFP* alpha feto-protein, *uE3* unconjugated Estriol

^a^First Trimester (T1) Biochemical Markers: PAPP-A, free-β hCG,PlGF, AFP, Inhibin A

^b^Second Trimester (T2) Biochemical Markers: AFP, uE3, hCG, PAPP-A, PlGF

^c^Through backward selection significant maternal characteristics for each case group were identified. Significant maternal characteristics for each case group include—All PE: age, weight, ethnicity, nulliparous; Preterm PE: age, weight, ethnicity, gravidity; Early-onset PE: age, gravidity; Gestational Hypertension: age, weight, ethnicity, nulliparous; Preterm Birth: nulliparous

Table 4 shows the estimated DR for PE, gestational hypertension and preterm birth at a FPR of 5%, 10%, and 20% using different biochemical marker combinations with and without maternal characteristics. Detection rates for preterm PE and early-onset PE were higher than for all PE cases. Adding maternal characteristics to any combinations yielded better DR values than maternal characteristics or biochemical marker combination alone. Detection rates for both gestational hypertension and preterm birth, using different biochemical marker combinations with and without maternal characteristics, were low.

**Table 4:** Model-predicted screening performance using maternal characteristics and different serum marker combinations

**Marker**	**Preeclampsia**			**Gestational hypertension**	**Preterm birth**
	**All**	**Preterm**	**Early-onset**		
***T1 Biochemical Markers ***^***a***^					
AUC	0.70 (0.654,0.753)	0.80 (0.734,0.867)	0.76 (0.635,0.890)	0.63 (0.593,0.663)	0.64 (0.588,0.685)
DR% (CI) for 5% FPR	23 (15.9,29.8)	25 (12.8,37.3)	35 (12.6,58.0)	13 (9.5,17.4)	17 (11.3,23.1)
DR% (CI) for 10% FPR	33 (25.1,40.6)	42 (27.7,55.6)	35 (12.6,58.0)	19 (14.8,24.0)	25 (18.1,31.6)
DR% (CI) for 20% FPR	55 (46.8,63.2)	73 (60.4,85.5)	65 (42.0,87.4)	35 (29.8,40.9)	40 (32.5,47.8)
***T2 Biochemical Markers ***^***b***^					
AUC	0.69 (0.620,0.755)	0.76 (0.645,0.873)	0.83 (0.608,1.000)	0.58 (0.531,0.638)	0.65 (0.581,0.725)
DR% (CI) for 5% FPR	23 (13.2,32.1)	29 (11.0,47.4)	50 (15.4,84.6)	12 (6.7,18.0)	19 (9.8,28.5)
DR% (CI) for 10% FPR	31 (20.2.41.1)	42 (21.9,61.4)	63 (29.0,96.0)	20 (13.1,26.9)	29 (18.6,40.2)
DR% (CI) for 20% FPR	44 (32.8,55.2)	58 (38.6,78.1)	88 (64.6,100.0)	28 (20.7,36.2)	38 (26.9,49.8)
***Maternal Characteristics Only ***^***c***^					
AUC	0.70 (0.661,0.745)	0.71 (0.632,0.779)	0.68 (0.558,0.806)	0.74 (0.707,0.771)	0.55 (0.510,0.594)
DR% (CI) for 5% FPR	18 (11.9,24.5)	15 (4.7,25.0)	18 (0,35.8)	22 (17.1,26.9)	7 (3.2,11.5)
DR% (CI) for 10% FPR	31 (23.2,38.3)	30 (16.7,42.9)	18 (0,35.8)	34 (28.7,39.9)	15 (9.6,21.1)
DR% (CI) for 20% FPR	45 (36.6.52.9)	47 (32.5,61.1)	41 (17.8,64.6)	54 (48.3,60.0)	25 (18.4,32.3)
***Maternal Characteristics ***^***c***^** + *T1 PlGF* + *T1 PAPP-A***					
AUC	0.78 (0.740,0.821)	0.83 (0.778,0.890)	0.82 (0.702,0.944)	0.75 (0.723,0.787)	0.61 (0.560,0.660)
DR% (CI) for 5% FPR	30 (22.2,37.6)	35 (21.0,48.6)	41 (17.8,64.6)	23 (18.2,28.4)	15 (9.3,20.9)
DR% (CI) for 10% FPR	42 (33.3,49.9)	52 (37.7,66.6)	65 (42.0,87.4)	39 (33.6,45.4)	21 (14.6,27.9)
DR% (CI) for 20% FPR	60 (51.7,68.1)	67 (53.8,80.9)	76 (56.3,96.6)	58 (52.0,63.8)	36 (28.5,44.1)
***Maternal Characteristics ***^***c***^** + *All T1 Biochemical Markers ***^***a***^					
AUC	0.79 (0.749,0.828)	0.84 (0.780,0.897)	0.84 (0.725,0.960)	0.76 (0.728,0.791)	0.64 (0.589,0.691)
DR% (CI) for 5% FPR	30 (22.2,37.6)	37 (23.0,50.9)	47 (23.3,70.8)	24 (18.6,28.8)	19 (12.8,25.6)
DR% (CI) for 10% FPR	45 (36.9,53.6)	59 (44.5,72.9)	71 (48.9,92.3)	40 (34.3,46.1)	29 (21.4,36.1)
DR% (CI) for 20% FPR	59 (50.9,67.4)	72 (58.7,84.8)	82 (64.2,100.0)	59 (52.7,64.6)	40 (31.8,47.7)
***Maternal Characteristics ***^***c***^** + *All T2 Biochemical Markers ***^***b***^					
AUC	0.78 (0.723,0.836)	0.80 (0.703,0.901)	0.83 (0.594,1.000)	0.76 (0.713,0.807)	0.70 (0.621,0.779)
DR% (CI) for 5% FPR	27 (17.2,37.6)	39 (19.2,59.0)	75 (45.0,100.0)	29 (20.8,37.3)	18 (7.7,27.4)
DR% (CI) for 10% FPR	38 (27.2,49.5)	48 (27.4,68.2)	75 (45.0,100.0)	40 (31.3,49.1)	37 (24.3,49.4)
DR% (CI) for 20% FPR	63 (51.9,74.1)	65 (45.8,84.7)	75 (45.0,100.0)	56 (47.4,65.4)	58 (45.1,70.7)

*DR* Detection Rate, *FPR* False Positive Rate, *AUC* Area under the Curve, *CI* 95% confidence interval

^a^First Trimester (T1) Biochemical Markers: PAPP-A, free-β hCG,PlGF, AFP, Inhibin A. Refer to Table 3 for specific markers used in each case group

^b^Second Trimester (T2) Biochemical Markers: AFP, uE3, hCG, PAPP-A, PlGF. Refer to Table 3 for specific markers used in each case group

^c^Significant maternal characteristics for each case group include- All PE: age, weight, ethnicity, nulliparous; Preterm PE: age, weight, ethnicity, gravidity; Early-onset PE: age, gravidity; Gestational Hypertension: age, weight, ethnicity, nulliparous; Preterm Birth: nulliparous

Table 5 gives the positive and negative LR and their 95% confidence intervals at a FPR of 5%, 10%, and 20% using different marker combinations for PE, gestational hypertension and preterm birth. For each case group, the highest positive LR and lowest negative LR were observed using maternal characteristics with second trimester serum markers, followed by maternal characteristics with first trimester serum markers. With the latter marker combination, at a FPR of 20%, there was a 4.1-fold increase in the odds of developing early-onset PE in women with a positive test result. A negative result would decrease the odds of having the condition by 4.6-fold. With the same marker combination, at the same FPR, the highest positive LR and lowest negative LR were observed for early-onset PE.

**Table 5:** The positive and negative likelihood ratios using maternal characteristics and different serum marker combinations

**Marker**	**Preeclampsia**						**Gestational hypertension**		**Preterm birth**	
	All		Preterm		Early-onset					
	LR + (CI)	LR- (CI)	LR + (CI)	LR- (CI)	LR + (CI)	LR- (CI)	LR + (CI)	LR- (CI)	LR + (CI)	LR- (CI)
**T1 Biochemical Markers**^**a**^										
5% FPR	4.53 (3.14,6.53)	0.81 (0.74,0.89)	4.95 (2.91,8.42)	0.79 (0.67,0.93)	6.99 (3.56,13.73)	0.68 (0.48,0.97)	2.66 (1.86,3.81)	0.91 (0.87,0.96)	3.40 (2.29,5.08)	0.87 (0.81,0.94)
10% FPR	3.27 (2.49,4.31)	0.75 (0.66,0.84)	4.15 (2.89,5.97)	0.65 (0.51,0.82)	3.52 (1.82,6.79)	0.72 (0.51,1.02)	1.94 (1.47,2.55)	0.90 (0.84,0.95)	2.47 (1.82,3.34)	0.84 (0.76,0.92)
20% FPR	2.75 (2.30,3.28)	0.56 (0.47,0.68)	3.64 (2.92,4.32)	0.34 (0.21,0.54)	3.23 (2.25,4.65)	0.44 (0.23,0.84)	1.77 (1.47,2.12)	0.81 (0.74,0.88)	2.00 (1.62,2.48)	0.75 (0.66,0.85)
**T2 Biochemical Markers**^**b**^										
5% FPR	4.53 (2.72,7.55)	0.81 (0.72,0.92)	5.83 (2.93,11.61)	0.75 (0.58,0.96)	10.00 (4.72,21.20)	0.53 (0.26,1.05)	2.40 (1.43,4.24)	0.92 (0.86,0.99)	3.74 (2.12,6.59)	0.85 (0.76,0.96)
10% FPR	3.07 (2.06,4.55)	0.77 (0.66,0.90)	4.17 (2.49,6.97)	0.65 (0.46,0.91)	6.25 (3.52,11.09)	0.42 (0.17,1.02)	2.00 (1.34,2.98)	0.89 (0.81,0.97)	2.84 (1.87,4.32)	0.79 (0.67,0.92)
20% FPR	2.20 (1.65,2.94)	0.70 (0.57,0.86)	2.92 (2.03,4.20)	0.52 (0.32,0.84)	4.38 (3.26,5.87)	0.16 (0.03,0.98)	1.42 (1.05,1.93)	0.89 (0.80,1.00)	1.86 (1.34,2.58)	0.78 (0.64,0.94)
**Maternal Characteristics Only**										
5% FPR	3.63 (2.42,5.44)	0.86 (0.80,0.93)	2.98 (1.46,6.08)	0.90 (0.80,1.01)	3.53 (1.23,10.05)	0.87 (0.70,1.08)	4.40 (3.26,5.95)	0.82 (0.77,0.88)	1.47 (0.80,2.68)	0.98 (0.93,1.02)
10% FPR	3.08 (2.32,4.08)	0.77 (0.69,0.86)	2.98 (1.88,4.72)	0.78 (0.65,0.94)	1.78 (0.63,5.03)	0.91 (0.73,1.14)	3.43 (2.76,4.25)	0.73 (0.67,0.80)	1.53 (1.03,2.29)	0.94 (0.88,1.01)
20% FPR	2.24 (1.82,2.75)	0.69 (0.60,0.80)	2.34 (1.70,3.22)	0.67 (0.51,0.87)	2.04 (1.15,3.63)	0.74 (0.50,1.10)	2.71 (2.34,3.12)	0.57 (0.50,0.65)	1.27 (0.95,1.69)	0.93 (0.85,1.03)
**Maternal Characteristics**^**c**^** + T1 PlGF + T1 PAPP-A**										
5% FPR	5.96 (4.28,8.29)	0.74 (0.66,0.82)	6.92 (4.43,10.83)	0.69 (0.56,0.85)	8.20 (4.48,15.01)	0.62 (0.42,0.92)	4.64 (3.43,6.27)	0.81 (0.76,0.86)	3.00 (1.94,4.65)	0.89 (0.83,0.96)
10% FPR	4.14 (3.24,5.29)	0.65 (0.56,0.75)	5.19 (3.80,7.09)	0.53 (0.39,0.72)	6.44 (4.41,9.41)	0.39 (0.21,0.75)	3.93 (3.20,4.83)	0.67 (0.61,0.74)	2.11 (1.50,2.98)	0.88 (0.80,0.95)
20% FPR	2.98 (2.52,3.52)	0.50 (0.41,0.62)	3.36 (2.69,4.20)	0.41 (0.27,0.62)	3.80 (2.88,5.04)	0.29 (0.13,0.69)	2.89 (2.51,3.33)	0.53 (0.46,0.61)	1.81 (1.43,2.29)	0.80 (0.70,0.90)
**Maternal Characteristics**^**c**^** + All T1 Biomarkers**^**a**^										
5% FPR	5.96 (4.28,8.29)	0.74 (0.66,0.82)	7.36 (4.78,11.32)	0.66 (0.53,0.83)	9.37 (5.44,16.16)	0.56 (0.36,0.87)	4.71 (3.49,6.36)	0.80 (0.75,0.86)	3.82 (2.58,5.65)	0.85 (0.79,0.92)
10% FPR	4.50 (3.57,5.69)	0.61 (0.52,0.71)	5.84 (4.41,7.74)	0.46 (0.33,0.65)	7.03 (5.01,9.86)	0.33 (0.16,0.68)	4.00 (3.26,4.91)	0.66 (0.60,0.73)	2.87 (2.14,3.84)	0.79 (0.71,0.88)
20% FPR	2.95 (2.49,3.49)	0.51 (0.42,0.63)	3.58 (2.92,4.40)	0.35 (0.22,0.56)	4.11 (3.24,5.23)	0.22 (0.08,0.62)	2.93 (2.55,3.36)	0.52 (0.45,0.60)	1.98 (1.59,2.48)	0.75 (0.66,0.86)
**Maternal Characteristics**^**c**^** + All T2 Biomarkers**^**b**^										
5% FPR	5.46 (3.39,8.81)	0.76 (0.66,0.88)	7.80 (4.32,14.08)	0.64 (0.46,0.89)	14.86 (9.04,24.42)	0.26 (0.08,0.87)	5.79 (3.84,8.74)	0.75 (0.66,0.84)	3.48 (1.84,6.56)	0.87 (0.77,0.98)
10% FPR	3.82 (2.68,5.46)	0.69 (0.57,0.82)	4.77 (2.97,7.66)	0.58 (0.39,0.86)	7.43 (4.74,11.63)	0.28 (0.08,0.92)	4.00 (2.96,5.41)	0.67 (0.57,0.77)	3.65 (2.46,5.42)	0.70 (0.58,0.86)
20% FPR	3.14 (2.51,3.92)	0.46 (0.34,0.63)	3.25 (2.34,4.51)	0.44 (0.25,0.76)	3.73 (2.45,5.70)	0.31 (0.09,1.04)	2.81 (2.28,3.47)	0.55 (0.44,0.67)	2.88 (2.23,3.74)	0.53 (0.39,0.72)

*LR (* +*)* Positive Likelihood Ratio, *LR (-)*: Negative Likelihood Ratio, *CI* 95% confidence Interval

^a^First Trimester (T1) Biochemical Markers: PAPP-A, free-β hCG,PlGF, AFP, Inhibin A. Refer to Table 3 for specific markers used in each case group

^b^Second Trimester (T2) Biochemical Markers: AFP, uE3, hCG, PAPP-A, PlGF. Refer to Table 3 for specific markers used in each case group

^c^Significant maternal characteristics for each case group include- All PE: age, weight, ethnicity, nulliparous; Preterm PE: age, weight, ethnicity, gravidity; Early-onset PE: age, gravidity; Gestational Hypertension: age, weight, ethnicity, nulliparous; Preterm Birth: nulliparous

**Fig. 2:**
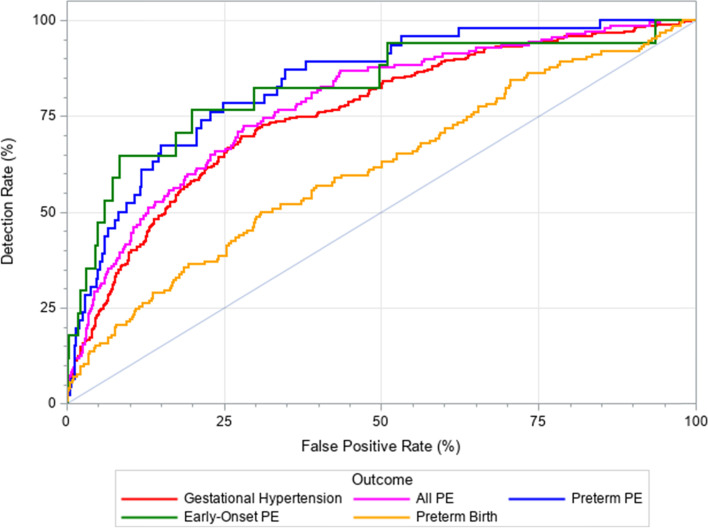
ROC curves for models including maternal characteristics and first trimester PAPP-A + PlGF for preterm delivery (AUC 0.61), gestational hypertension (AUC 0.75), all preeclampsia (PE) (AUC 0.78), preterm preeclampsia (AUC 0.83), and early-onset preeclampsia (AUC 0.82) cases

Figure 2 demonstrates the ROC curves for PE, gestational hypertension and preterm birth using maternal characteristics and PlGF and PAPP-A in the first trimester. The area under the ROC curve (AUC) was largest among preterm PE (0.83) and early-onset PE (0.82) cases followed by all PE (0.78), gestational hypertension (0.75) and preterm birth cases (0.61). The best screening performance for a FPR < 25% was seen for early-onset PE followed by preterm PE and all PE.

## Discussion

Our study assessed whether first and second trimester biochemical markers used in prenatal aneuploidy screening could accurately identify pregnancies at risk of developing adverse pregnancy outcomes, mainly PE. We found that the combination of maternal characteristics with biochemical markers in either trimester can provide reasonable accuracy in identifying women at risk of developing preterm and early-onset PE but not women at risk for gestational hypertension or preterm birth. PAPP-A and PlGF in the first and second trimesters were the biochemical markers most reliably associated with and predictive of adverse pregnancy outcomes, particularly early-onset PE.

Our results have confirmed previously reported associations between individual biochemical markers and PE and preterm birth [7–9]. Similar to previous studies, we found the performance of PE prediction to be improved by using multiple biochemical markers in combination with maternal characteristics [5, 11, 12].The most commonly used marker combination of first trimester PAPP-A and PlGF along with maternal characteristics can predict 65% and 76% of early-onset PE with a FPR of 10% and 20% respectively. Second trimester biochemical markers alone can identify 88% of the pregnancies at risk of developing early-onset PE with a FPR of 20%. However, this will require the addition of PAPP-A and PlGF which are not currently used in second trimester aneuploidy screening. In addition, we found that the increase of PlGF with gestational age was smaller in women affected by PE compared to the control group, particularly in those affected by early-onset PE.

Low first trimester PAPP-A and PlGF have been associated with adverse pregnancy outcomes, especially PE in multiple studies [5, 11–13]. The case-control study by Keikkala et al. (2016) investigating maternal serum samples from 8-13 weeks of gestation found lower median MoM values for PlGF and PAPP-A in women with PE compared to controls [13]. Consistent with our study, the lowest median MoM values were seen in cases with preterm and early-onset PE. As with previous studies, our study found median first trimester AFP MoM values to be higher and median free β-hCG MoM values to be lower in women with PE, although the significance of these changes was smaller in comparison to those of PAPP-A and PlGF [8, 9]. The previously described association between first trimester inhibin A and PE was not confirmed in our study [14, 15].

Second trimester PlGF and PAPP-A median MoM values were lower in our study than those reported by other studies [9, 16]. However, this was mainly true for PlGF and not significant for PAPP-A in women with preterm or early-onset PE, likely due to the small number of pregnancies in this group. We found an increase in total hCG in PE pregnancies, as reported in previous studies [17]. In contrast, no change in Inhibin A was seen in our study although reported as an optimal second trimester marker by others [18]. We suspect this is due to the small number of early PE pregnancies involved in our study. Lastly, the change in the first and second trimester markers in gestational hypertension and preterm birth cases was consistent with other studies in general although the magnitude of the change was variable [19, 20].

In recent years, the results of several large clinical trials have suggested that the performance of PE screening can be improved by using multiple biochemical markers together with maternal characteristics and biophysical markers [5, 12, 21]. A prospective study by Akolekar et al. on 58,884 singleton pregnancies at 11–13 weeks reported a 50.5% DR at a 10% FPR with maternal characteristics alone for early-onset PE, 74.3% with the addition of PlGF and PAPP-A and 89.7% with the addition of biophysical markers, MAP and UTPI [5]. The DR improved to 96.3% when maternal characteristics and biochemical and biophysical markers were combined [12].Similarly, in a prospective study of 35,948 singleton pregnancies at 11-13 weeks’ gestation, O’Gorman et al. found that combined screening obtained a DR of 75% and 47% at a 10% FPR for preterm PE and term PE pregnancies, respectively [12]. When such modelling combinations were applied to the ASPRE trial, similar DR values were observed. First-trimester screening for preterm PE with a risk cut-off of 1 in 100 detected 76.7% of preterm PE and 43.1% of term PE pregnancies, at a screen-positive rate of 10.5% and a FPR of 9.2% [21].

While the best performance can be achieved by combining multiple variables (e.g., maternal characteristics, medical and family history, biochemical and biophysical markers), MAP and especially UTPI might not be readily accessible, especially for women in remote areas. If the combination of maternal characteristics and biochemical markers in the first trimester can predict the risk of PE with reasonable accuracy, the current aneuploidy screening could be expanded to include PE screening to identify women at increased risk of developing PE, with biophysical markers to be followed as a second line or contingent screen. In our study, the PE screening performance was comparable to previous studies when using maternal characteristics in combination with first trimester PAPP-A and PlGF. At a FPR of 20%, the DR was 76%, 67% and 60% for early-onset PE, preterm PE and all PE, respectively. This suggests that for 20% of women who screen positive for early-onset PE, a contingent PE screening strategy using MAP and UTPI followed right after the biochemical screen can predict up to 76% of early-onset PE. As with the one-time screening approach, our contingent screen can yield final PE screening results before 16 weeks of gestation in order to initiate prophylactic therapy with Aspirin.

Although a contingent approach requires minimal change to the current screening program, there is a chance of missing the optimal time window for prophylactic therapy if there is a delay in measuring MAP and UTPI. Based on our local experience of utilizing MAP and UTPI, the gestational age at first trimester screening and the test turnaround time, we anticipated a substantial proportion of women could receive the final result before 16 weeks of gestation. When timely MAP and UTPI examinations are not feasible, maternal characteristics and first trimester biochemical markers only can also be used as a ‘mini’ PE screening test to identify women who might benefit from prophylactic therapy. Nevertheless, the addition of secondary MAP and UTPI would improve the test accuracy by reducing its FPR.

First trimester screening aims to identify those at risk of PE within the target window for treatment with Aspirin. Second trimester screening, on the other hand, is useful for patient triage, by identifying pregnancies that need close surveillance and more urgent medical attention. For example, numerous studies have focused on second trimester PlGF or soluble fms-like tyrosine kinase-1 (sFlt-1)/PlGF ratio [22]. In our study, in addition to the PE screening performance using second trimester markers, we compared the changes in biochemical markers between the first and second trimester samples. For women who developed PE, the increase in PlGF concentrations was significantly smaller compared to controls. These results have been confirmed by the findings from other studies [23–25]. Theoretically, for women who are identified to be at high risk for PE in the first trimester, a repeat test for PlGF and PAPP-A in the second trimester might provide useful information which can be used for risk modification. Nevertheless, a calculation of biomarker changes between trimesters did not yield a better prediction for PE than using second trimester markers alone in our study. Further investigation of this trend may provide clinicians with valuable information for monitoring and early detection of high-risk women.

In our study, the preterm birth screening performance was less optimal than previously reported [20]. A possible reason for the lower DR is that unlike some previous studies, our preterm birth group excluded all women with PE and gestational hypertension. Incomplete maternal characteristics data may have also contributed to the lower-than-expected DR values for preterm birth.

Our study assessed the accuracy of a PE screening approach originating from established multiple marker aneuploidy screening. For screening programs that use primary cffDNA test, a separate system that involves clinical, biochemical and ultrasound expertise would be required in order to introduce PE screening. While the patient pathway and screening process might be different in a program specifically designed for PE screening, our findings relating to how biochemical markers may assist in identifying women at risk of developing PE can be generalized. Although an ideal PE screening test should incorporate all test components, the contingent approach we have described in this study provides an option to programs that are unable to offer MAP and UTPI to all screened women due to limited resources.

The strengths of our study include the identification of cases and controls from a routine unselected screening population, representing a true sample of women undergoing prenatal screening in Ontario. Also unique to our study is the availability of both first and second trimester serum samples for most cases and controls. Having samples from both trimesters enabled us to investigate the change in biochemical markers between the first and second trimesters, which might provide additional information for PE screening and monitoring. The limitations of this study include the transfer of some women to other obstetrical centres, potentially lowering the incidence of PE in our population. Additionally, our local population includes a greater proportion of women of Asian ancestry compared with other studies. Varying ethnicity between studies may impact PE prevalence; in particular, a lower prevalence of PE among Asian women has been noted previously [26]. Our study lacked complete information on all maternal characteristics and as such, the accuracy of PE screening using maternal characteristics cannot be directly compared to studies where maternal characteristics were explicitly collected for PE screening. However, the contribution of biochemical markers and overall PE screening performance using the combination of maternal characteristics and biochemical markers were consistent with previous studies. In a real-life clinical setting, it will be possible to collect all maternal characteristics missed in this study. Since information on MAP and UTPI was not available to our study, we were not able to assess the final performance of a contingent PE screening strategy. The contingent approach we described in this study lacked validation. Implement of this screening strategy would require close collaborations of a multidisciplinary team. In addition to timely multiple marker screening, MAP and UTPI results, a quality assurance scheme for MAP and UTPI should also be in place before the test can be adopted for clinical utilization. Two studies are currently underway in our program to validate the performance of the first-tier PE screening in a different population, and to assess the feasibility, and patient and provider acceptance of the contingent screening approach. Nevertheless, we achieved our goal of assessing the first-tier of a contingent screening strategy, one that provides a reasonable performance, warranting expansion of current aneuploidy screening to include preeclampsia.

## Conclusions

Our study, based on a routine aneuploidy screening, showed that the combination of maternal characteristics and first trimester serum PAPP-A and PlGF can provide reasonable performance for PE screening. If our study results can be validated by prospective studies, the current aneuploidy screening program can be expanded to include identification of women at risk of developing PE, particularly early-onset PE. As a contingent strategy, it can provide first-tier PE screening with minimal associated costs and minimal change to the current workflow.

## Data Availability

All data generated or analysed during this study are included in this published article.

## Abbreviations

AFP: α-Fetoprotein
AUC: Area Under the Curve
BMI: Body mass index
cfDNA: Cell-free fetal DNA
DR: Detection rate
EDD: Expected date of delivery
FPR: False positive rate
hCG: Human chorionic gonadotrophin
IUGR: Intrauterine growth restriction
IVF: In vitro fertilization
MAP: Mean arterial blood pressure
MoM: Multiple of the median
NT: Nuchal translucency
PAPP-A: Pregnancy-associated plasma protein A
PE: Preeclampsia
PlGF: Placental growth factor
QUAD: Second trimester serum screening
ROC: Receiver operating characteristic
sFlt-1: Soluble fms-like tyrosine kinase-1
T1: First trimester
T2: Second trimester
uE3: Unconjugated estriol
UTPI: Uterine artery pulsatility index
